# SG-APSIC1096: Changes in resistance patterns of “ESKAPE” pathogens to azithromincin and levofloxacin in Yogyakarta

**DOI:** 10.1017/ash.2023.84

**Published:** 2023-03-16

**Authors:** Rizka Asdie, Faisal Heryono, Ika Puspitasari, Tri Wibawa, Abu Tholib Aman, Andaru Dahesihdewi, Ludang Rizki Pradhipta

**Affiliations:** Perdalin Cabang Yogyakarta, Dr Sardjito General Hospital, Yogyakarta, Indonesia; Perdalin Cabang Yogyakarta, Dr Sardjito General Hospital, Yogyakarta, Indonesia; Faculty of Pharmacy of Gadjah Mada University, Yogyakarta, Indonesia; Academic Hospital of Gadjah Mada University, Yogyakarta, Indonesia; Soeradji Tirtonegoro Hospital Klaten, Central Java, Indonesia; Perdalin Cabang Yogyakarta, Dr Sardjito General Hospital, Yogyakarta, Indonesia; PKU Muhammadiyah Hospital, Yogyakarta, Indonesia

## Abstract

**Objectives:** Bacterial coinfection occurred in 3.5% of COVID-19 patients, and secondary bacterial infection occurred in 14.3% of patients. In Indonesia, one of the guidelines for COVID-19 therapy is to administer azithromycin 500 mg per 24 hours for mild and moderate cases and azithromycin 500 mg per 24 hours and levofloxacin 750 g per 24 hours for severe cases with suspected secondary bacterial infection. At the beginning of the pandemic, many antibiotics were used, even without proven or suspected bacterial infection. We sought to determine changes in the resistance of “ESKAPE” bacteria (ie, *Enterococcus faecium*, *Staphylococcus aureus*, *Klebsiella pneumoniae*, *Acinetobacter baumannii*, *Pseudomonas aeruginosa*, and *Enterobacter* spp) to the antibiotics levofloxacin and azithromycin prior to and during the COVID-19 pandemic. **Methods:** The study was conducted retrospectively by examining the culture and sensitivity test results of “ESKAPE” bacteria to levofloxacin and azithromycin antibiotics in 2019 (before the pandemic) and April 2020–April 2021 (during the pandemic) in 4 hospitals in Yogyakarta. The number of samples represents all cultures completed within the specified period to detect antibiotic sensitivity patterns. **Results:** In a top referral hospital, resistance to levofloxacin and azithromycin increased significantly for *E. faecium* and *P. aeruginosa*, but at a private hospital, an increase in resistance to azithromycin and levofloxacin occurred for *A. baumannii* and for *Enterobacter* spp and resistance to levofloxacin increased significantly. At an academic hospital, there was a considerable decrease in *S. aureus* and *E. faecium* resistance to levofloxacin and azithromycin. At the government hospital, *S. aureus*, *K. pneumoniae*, *P. aeruginosa*, *Acinetobacter baumannii*, and *Enterobacter* spp developed resistance to levofloxacin. **Conclusions:** Resistance to azithromycin and levofloxacin by different ESKAPE bacteria increased on average during the COVID-19 pandemic.

